# Association Between One’s Preferred Film Genres and Personality Traits: A Cross-Sectional Study

**DOI:** 10.7759/cureus.84683

**Published:** 2025-05-23

**Authors:** Arjun S Menon, Chaitra M. S., Srinivasulu Naidu, Naladala Disha Chowdary

**Affiliations:** 1 Department of Internal Medicine, Shri Atal Bihari Vajpayee Medical College and Research Institute, Bengaluru, IND; 2 Department of Physiology, Shri Atal Bihari Vajpayee Medical College and Research Institute, Bengaluru, IND; 3 Department of Obstetrics and Gynecology, Shri Atal Bihari Vajpayee Medical College and Research Institute, Bengaluru, IND

**Keywords:** agreeableness, big five inventory, conscientiousness, extraversion, film genres, neuroticism, openness, personality traits

## Abstract

Objective: This study explores the correlation between college students' film genre preferences and their personality traits, as defined by the Big Five theory. Additionally, it seeks to evaluate the potential application of these correlations in hiring and screening processes.

Method: A total of 300 college students aged 20-23 participated in the study. Participants completed a demographic survey, ranked their preferred film genres, and responded to the Big Five inventory questionnaire. Data were analyzed using correlation and regression tests to determine the relationship between film genre preferences (independent variable) and personality traits (dependent variable), with a significance level set at p < 0.05.

Results: The analysis revealed significant correlations between preferred film genres and personality traits for all genres except comedy. Adventure films showed a positive correlation with extraversion but negative with conscientiousness and neuroticism. Drama correlated positively with conscientiousness and neuroticism, while romance linked to neuroticism and openness. Horror films were positively associated with extraversion and agreeableness, and mystery films correlated with openness.

Conclusion: The findings suggest that college students' film genre preferences significantly correlate with their personality traits. These correlations may have implications for personality assessments in various contexts, including hiring practices. However, further research is necessary to generalize these results beyond the studied population.

## Introduction

Personality is defined as the amalgamation of characteristics or qualities that shape an individual's distinctive character. According to the Big Five theory of personality traits, examples include openness, conscientiousness, extraversion, agreeableness, and neuroticism. Conversely, a genre is defined as a style or category within art, music, or literature. Common film genres encompass action, adventure, comedy, and drama.

Numerous studies have suggested that one's preferred film genre can offer insights into their personality traits. For instance, Mohamed Naseef and Neelakandan, in their research, identified positive correlations between extraversion and the film genres of comedy and romance, while agreeableness and conscientiousness correlated positively with the romance genre [1]. These findings hold potential implications, particularly in screening assessments, where an individual's personality can be evaluated based on their cinematic preferences. Furthermore, with additional research, such insights could inform the selection of film genres conducive to the cognitive development of younger audiences.

It was observed in 2010 that youth collectively consumed approximately 10 hours and 45 minutes of media content daily [2]. Given the escalating prevalence of digital device usage among the youth, the findings of such research could prove invaluable in understanding media consumption patterns and their implications.

The Big Five Inventory (BFI) is a widely recognized tool developed to assess the principal personality domains in adults: extraversion, agreeableness, conscientiousness, neuroticism, and openness to experience. This inventory has demonstrated reliability and validity in numerous psychological studies [3] and is instrumental in understanding how these traits influence behaviors, including preferences for film genres and social media engagement.

Openness

High levels of openness are associated with a greater appreciation for novelty and diverse experiences. Research indicates that individuals scoring high in openness tend to prefer genres such as documentaries and science fiction, reflecting their curiosity and desire for intellectual stimulation [4].

Conscientiousness

Conscientious individuals often display high levels of organization and responsibility. Studies suggest they may gravitate toward structured narratives in genres like drama [5]. This trait also correlates with careful curation of social media content, where conscientious users prioritize quality and thoughtful engagement [6].

Extraversion

Extraversion is characterized by sociability and emotional expressiveness. Recent research shows that extraverts are more likely to prefer comedic and romantic films, which resonate with their social nature and need for emotional engagement [7].

Agreeableness

Those high in agreeableness exhibit prosocial behaviors and are often drawn to uplifting narratives. This trait has been linked to positive interactions on social media, where agreeable individuals tend to share supportive and altruistic content [8].

Neuroticism

Neuroticism is associated with emotional instability and influences media preferences by drawing individuals toward genres that reflect their emotional experiences, such as drama and romance [9]. Neurotic individuals might also engage in social media comparisons that exacerbate feelings of anxiety and insecurity.

This framework provides a comprehensive understanding of how personality traits relate to film genre preferences and social media behaviors, reinforcing the significance of these traits in personal and social contexts.

Despite the growing body of literature linking personality traits to preferences in media consumption, there remains a notable gap in understanding how these correlations can be systematically applied, particularly in the context of hiring and screening processes. This study aims to correlate individuals’ film genre preferences with their personality traits as defined by the BFI. By doing so, we seek to establish a foundation for utilizing cinematic preferences as a supplementary assessment tool in personality evaluation. Given the implications of personality traits in both personal and professional settings, our research could enhance screening methods by providing additional insights into candidates’ psychological profiles. This investigation not only contributes to the existing literature by exploring the nuanced relationships between personality and film preferences but also addresses practical applications in recruitment strategies, thereby bridging the gap between theoretical research and real-world implications.

## Materials and methods

Inclusion criteria

Inclusion criteria are as follows: 1) students aged 20-23 years currently enrolled at a university. This specific age range was selected for practical reasons, including ease of access to participants and the relative consistency of psychological development in early adulthood, and 2) students who can read and understand English, as all questionnaires used in the study were in English.

Exclusion criteria

Exclusion criteria include the following: 1) students currently taking any kind of medication for physical or mental health conditions, as medication side effects may influence personality expression, and 2) students who do not have a preferred film genre (i.e., those who do not consistently prefer one genre over others or who regularly watch movies across all genres without clear preference).

Study population and demographic

Students aged 20-23 years residing in India were selected. Participants were from diverse educational and socioeconomic backgrounds. The population was restricted to Indian students for logistical feasibility and to ensure consistent language and cultural context. The inclusion and exclusion criteria were strictly adhered to. While this narrow age range may limit the generalizability of the findings, it was selected to ensure uniformity and accessibility. Future studies should consider broader demographic groups to validate the findings across different life stages and populations.

Sample size calculation

The sample size (n) is calculated according to the formula: n = z^2^ * p * (1 - p) / e^2^, where z is 1.96 for a confidence level (α) of 95%, p is the proportion (expressed as a decimal; p = 0.11), and e is the margin of error (e = 0.05). n = 1.96^2^ * 0.11 * (1 - 0.11) / 0.052 n = 0.3761 / 0.0025 = 150.437 n ≈ 151

The sample size is equal to 151. However, 300 participants were included in the study to exceed the minimum required size and improve the validity of the findings.

Study parameters

All participants were of Indian descent and aged between 20 and 23. The final sample consisted of 300 students, with a mean age of 22.4 years, comprising 176 women and 124 men from varied socioeconomic backgrounds.

Study procedure

Participants were recruited in person and informed of the study's purpose and procedures. Ethical clearance was obtained from the institutional review board, and all participants provided written informed consent.

Each participant provided demographic information, including name, age, sex, educational background, and daily screen time. Participants were asked about their preferred film genres. Those with a clear preference were asked to rank six genres (adventure, drama, comedy, romance, horror, and thriller) from 1 (most preferred) to 6 (least preferred). Participants without a clear preference were excluded.

Subsequently, participants completed the BFI, a 44-item questionnaire that assesses the five major personality traits: extraversion, neuroticism, conscientiousness, openness, and agreeableness. Each item was rated on a 5-point Likert scale (1 = strongly disagree to 5 = strongly agree). The BFI is a validated, open-access tool widely used in personality research [10]. See the Appendix for the full questionnaire used in the study.

Statistical analysis

Data analysis was conducted using Microsoft Excel (Microsoft Corporation, Redmond, WA), specifically employing the ToolPak extension for statistical computations. Correlation and regression tests were applied to examine the relationship between film genre preference (independent variable) and Big Five personality traits (dependent variables).

The decision to treat film genre preference as the independent variable was based on our hypothesis that media preferences can reflect deeper psychological characteristics. This is supported by prior literature suggesting that personality influences media consumption patterns [11,12]. However, we acknowledge the bidirectional nature of this relationship and suggest future studies may treat personality as the predictor variable to examine its influence on genre preference.

A p value of <0.05 was considered statistically significant. A correlation coefficient of ≥0.25 (absolute value) was used to identify a meaningful association between personality traits and genre preferences.

## Results

A p value of less than 0.05 (i.e., a correlation coefficient with absolute value more than 0.25) indicated a significant correlation between one’s preferred film genre and one’s personality trait. The values Table 1 are the correlation coefficients.

**Table 1:** Correlation coefficients derived when the film genres were correlated with the Big Five personality traits

Variables	Extraversion	Agreeableness	Conscientiousness	Neuroticism	Openness	Adventure	Drama	Comedy	Romance	Horror	Mystery
Extraversion	1.00	-	-	-	-	-	-	-	-	-	-
Agreeableness	0.68	1.00	-	-	-	-	-	-	-	-	-
Conscientiousness	-0.15	0.39	1.00	-	-	-	-	-	-	-	-
Neuroticism	-0.62	-0.34	0.28	1.00	-	-	-	-	-	-	-
Openness	-0.48	-0.73	-0.31	0.42	1.00	-	-	-	-	-	-
Adventure	0.30	-0.01	-0.45	-0.28	0.04	1.00	-	-	-	-	-
Drama	-0.48	-0.19	0.50	0.52	0.15	-0.39	1.00	-	-	-	-
Comedy	-0.12	0.02	0.07	0.10	0.00	-0.12	-0.05	1.00	-	-	-
Romance	-0.49	-0.41	0.06	0.34	0.34	-0.40	0.26	0.08	1.00	-	-
Horror	0.55	0.71	0.30	-0.31	-0.61	-0.12	-0.17	-0.28	-0.55	1.00	-
Mystery	-0.03	-0.37	-0.50	-0.17	0.30	0.05	-0.50	-0.21	-0.08	-0.35	1.00

**Figure 1:**
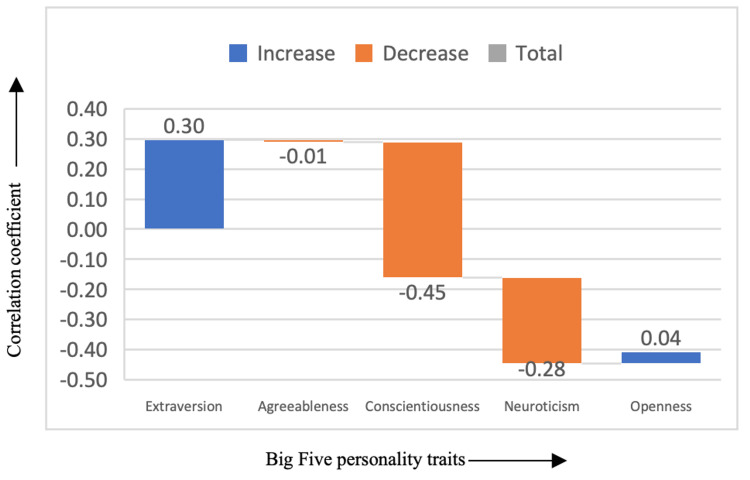
Correlation between adventure genre and the Big Five personality traits

The following are the graphs indicating how each genre corresponds to each of the Big Five personality traits. In Figure 1, the adventure genre showed a significant positive correlation with extraversion (correlation coefficient = 0.30), suggesting that individuals who prefer adventure films tend to be more extraverted, outgoing, and sociable. Conversely, adventure films had a negative correlation with conscientiousness (correlation coefficient = -0.45) and neuroticism (correlation coefficient = -0.28). This indicates that people who enjoy adventure films are less likely to display high levels of conscientiousness (a trait linked to organization and dependability) and may be more emotionally stable, with lower levels of neuroticism (emotional instability or anxiety). This pattern reflects the adventurous and spontaneous nature of individuals who gravitate toward action-packed genres that prioritize excitement and exploration over emotional or psychological depth.

**Figure 2:**
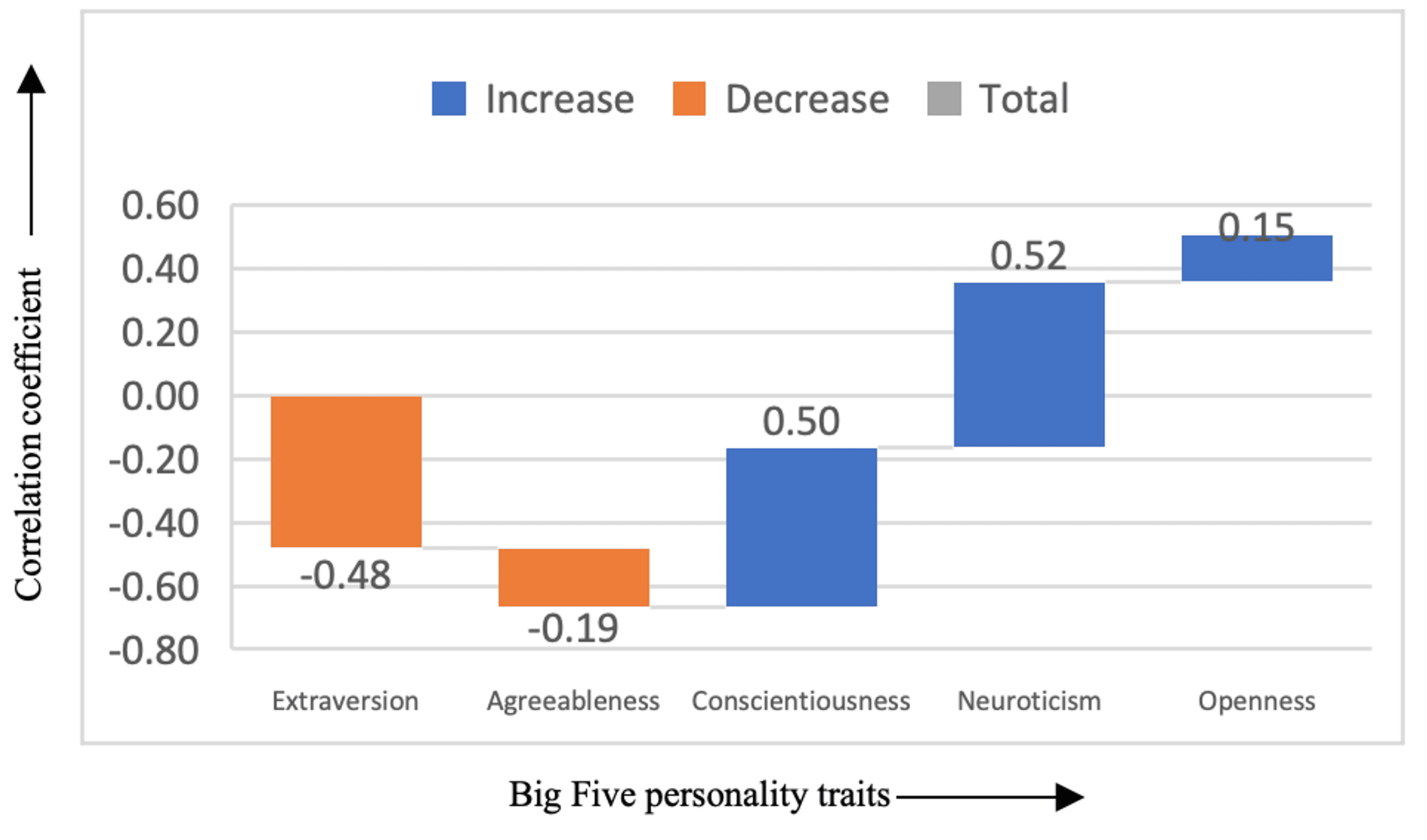
Correlation between drama genre and the Big Five personality traits

In Figure 2, the drama genre demonstrated a positive correlation with both conscientiousness (correlation coefficient = 0.50) and neuroticism (correlation coefficient = 0.52), suggesting that individuals who favor dramas may possess a higher degree of conscientiousness and emotional sensitivity. Drama lovers are likely to have a more structured approach to life (evidenced by conscientiousness) while also being more emotionally reactive or prone to stress (evidenced by neuroticism). Interestingly, drama films were negatively correlated with extraversion (correlation coefficient = -0.48), indicating that those who prefer dramas may be less extroverted or more introverted, finding emotional and intellectual stimulation in more serious, reflective content rather than in high-energy social scenarios.

**Figure 3:**
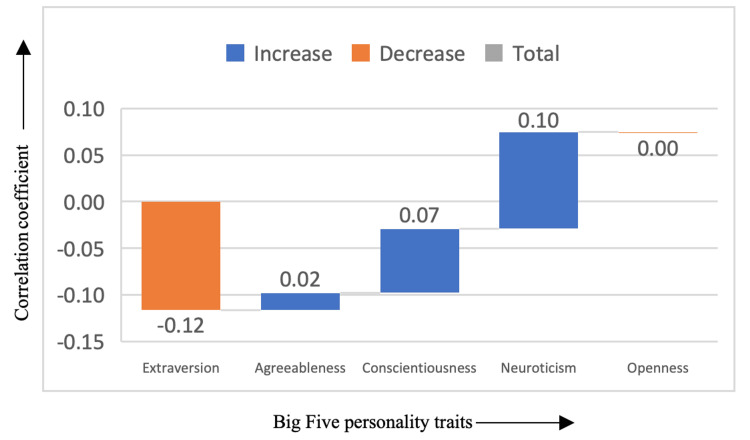
Correlation between comedy genre and the Big Five personality traits

In Figure 3, the comedy genre showed no significant correlations with any specific personality trait (correlation coefficient < |0.25|), suggesting that it appeals to a broad range of individuals regardless of their personality profile. Comedy films have a universal appeal, often offering lighthearted entertainment that transcends the specific emotional or psychological traits commonly associated with other genres. The lack of a significant relationship between comedy and personality traits implies that people of all personality types enjoy humor, making it less useful as an indicator of one's psychological makeup.

**Figure 4:**
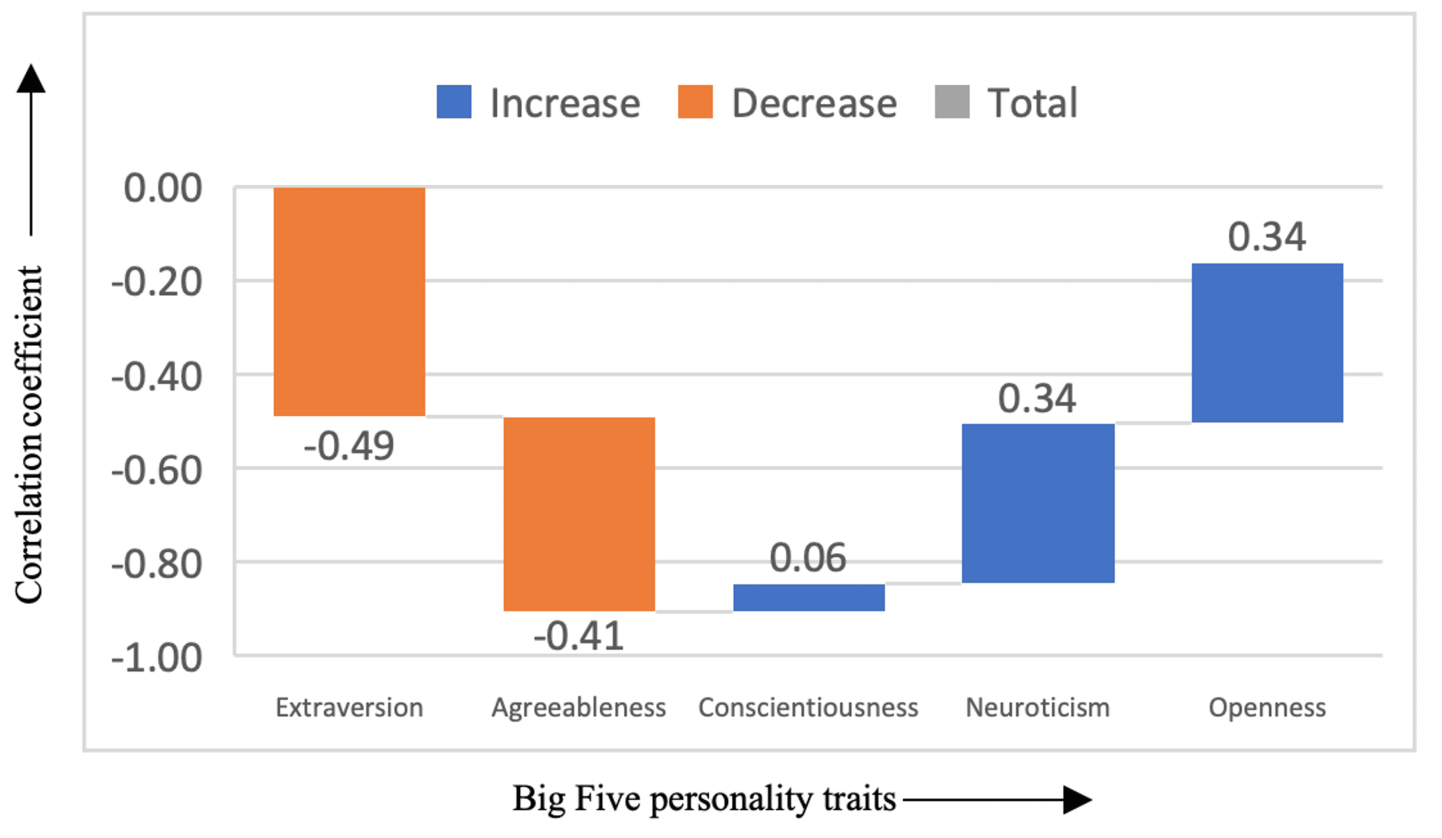
Correlation between romance genre and the Big Five personality traits

In Figure 4, the romance genre revealed a significant positive correlation with neuroticism (correlation coefficient = 0.34) and openness (correlation coefficient = 0.34), as well as a negative correlation with extraversion (correlation coefficient = -0.49) and agreeableness (correlation coefficient = -0.41). Those who prefer romance films tend to be more emotionally reactive (higher neuroticism) and open to new experiences, possibly due to the genre's focus on emotional connection, love, and relationships. Additionally, the negative correlation with extraversion suggests that romance film enthusiasts may be less sociable or outgoing, preferring the emotional intimacy and personal connection found in romantic narratives. The negative correlation with agreeableness suggests a potential for lower levels of cooperativeness, as romance films often focus on complex personal dynamics that may not always align with harmonious social interactions.

**Figure 5:**
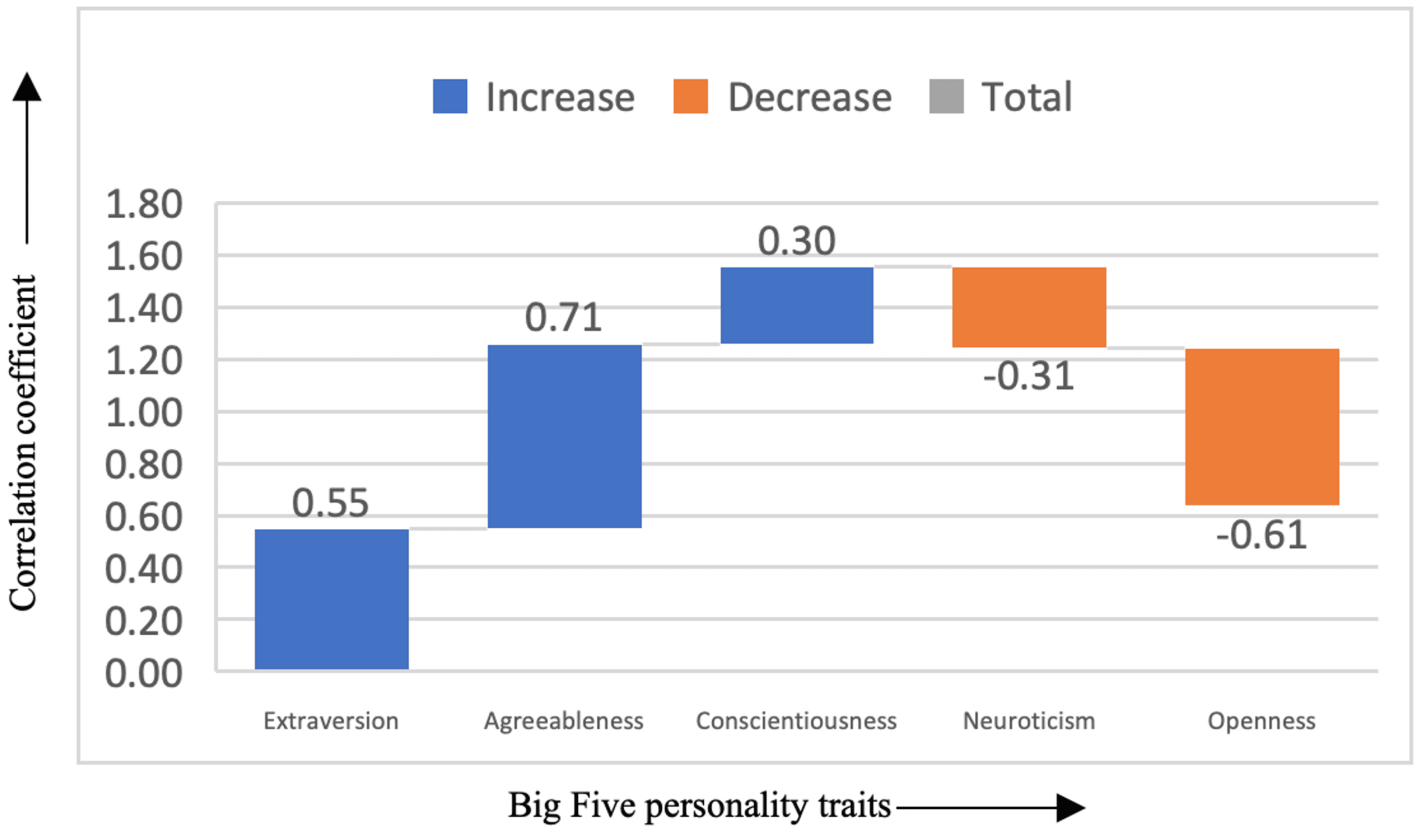
Correlation between horror genre and the Big Five personality traits

In Figure 5, the horror genre showed a positive correlation with extraversion (correlation coefficient = 0.55), agreeableness (correlation coefficient = 0.71), and conscientiousness (correlation coefficient = 0.30), while demonstrating negative correlations with neuroticism (correlation coefficient = -0.31) and openness (correlation coefficient = -0.61). This indicates that individuals who enjoy horror films are likely to be more extroverted, cooperative, and responsible, possibly because horror films often involve group settings and shared experiences (such as watching with others). Despite the intense and fear-inducing content, these individuals may not experience the same level of emotional instability (lower neuroticism) as others. The negative correlation with openness suggests that horror film fans may be less open to unconventional or abstract experiences, possibly preferring the straightforward thrill of fear-based narratives over more complex or cerebral genres.

**Figure 6:**
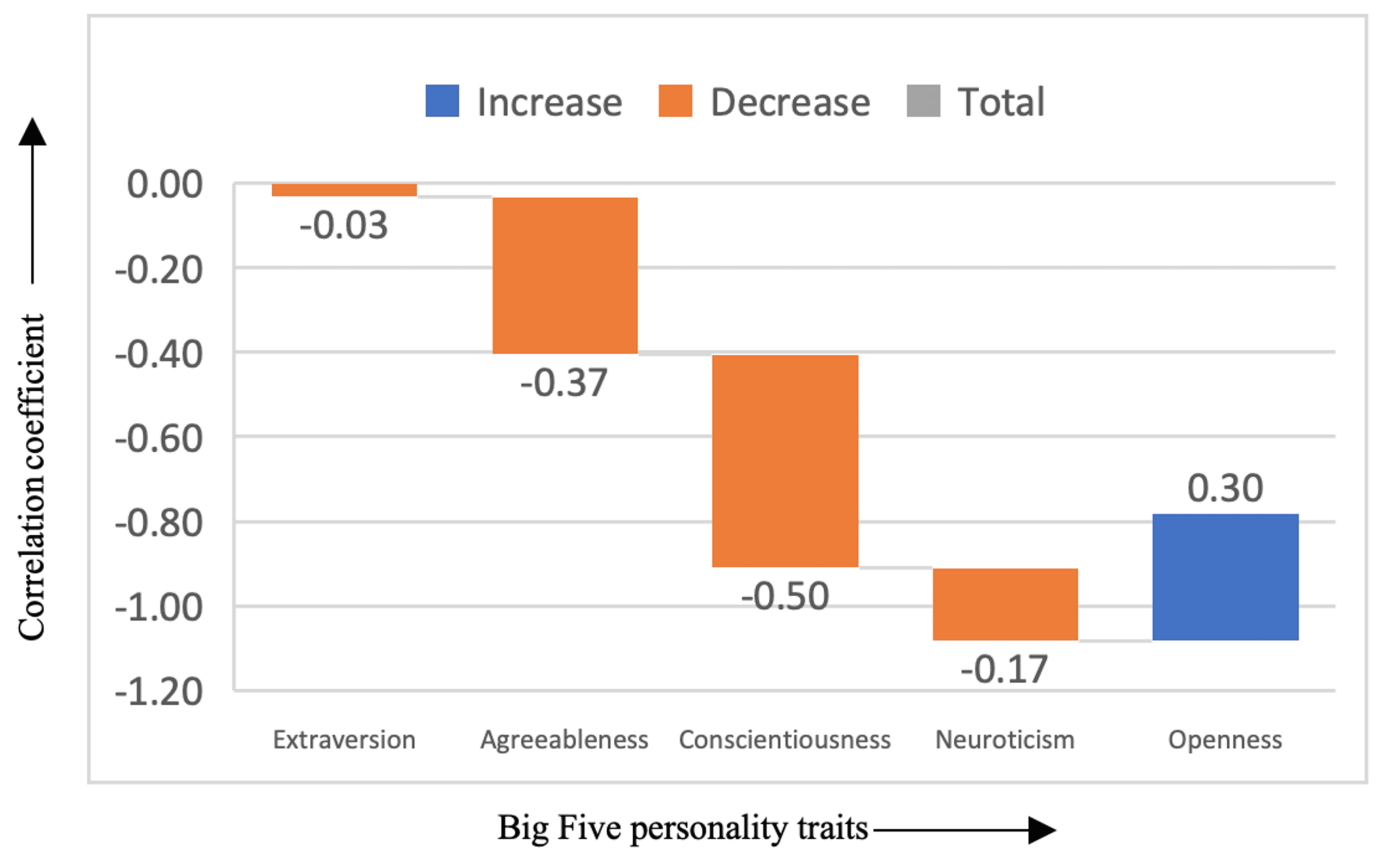
Correlation between mystery genre and the Big Five personality traits

In Figure 6, the mystery genre demonstrated a significant positive correlation with openness (correlation coefficient = 0.30), indicating that those who prefer mystery films tend to be more open to new ideas, experiences, and intellectual challenges. Mystery films, which often involve solving puzzles and uncovering hidden truths, may appeal to individuals who enjoy cognitive engagement and exploring the unknown. However, mystery films were negatively correlated with agreeableness (correlation coefficient = -0.37) and conscientiousness (correlation coefficient = -0.50), suggesting that mystery lovers may be less likely to prioritize social harmony and personal responsibility, perhaps due to the genre's emphasis on individual problem-solving and sometimes morally ambiguous situations.

**Figure 7:**
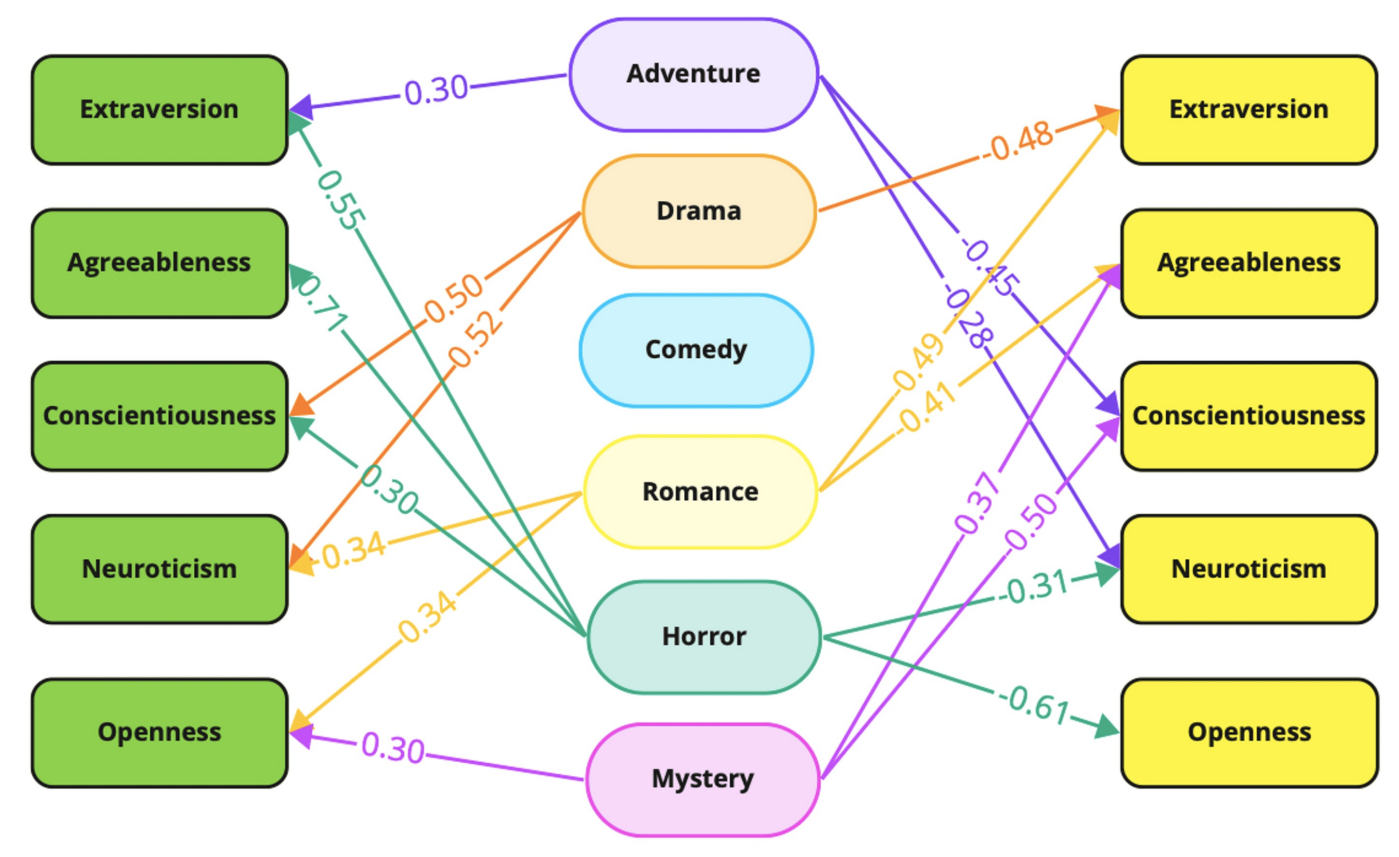
Representation of positive and negative correlations between the film genres and Big Five personality traits. The numbers on the arrows denote the correlation coefficients Image credits: This is an original image created by the author Arjun S. Menon

A pictorial representation of the correlations, shown in Figure 7, clearly maps the positive and negative relationships between film genres and personality traits. Each arrow is labeled with the correlation coefficient, visually illustrating the strength and direction of the correlation. These findings provide valuable insights into how film preferences can be indicative of underlying personality characteristics, confirming that an individual's choice of cinematic content may reflect broader psychological patterns.

## Discussion

The findings of this study underscore a significant relationship between college students' film genre preferences and their personality traits, as conceptualized by the Big Five theory. Our results suggest that individuals who prefer adventure films tend to exhibit higher levels of extraversion, while those inclined toward drama films display stronger conscientiousness and neuroticism. These associations are consistent with previous research that links certain personality traits with specific media preferences. For example, prior studies have shown that extraverts are more likely to enjoy action and adventure films, which align with their sociable and energetic traits [13,14]. Additionally, drama films have been shown to appeal to individuals who score high in neuroticism, which is characterized by emotional sensitivity and reactivity [12,13]. Horror film fans may lean toward being more extroverted, while mystery film fans may crave newer experiences [15,16]. Our findings similarly demonstrate that film genre preferences are not merely recreational choices but may reflect deeper psychological patterns that could be useful in personality assessments.

While this study provides valuable insights into the relationship between film genre preferences and personality traits, it is important to consider several limitations. First, the sample is limited to college students aged 20-23, which may restrict the generalizability of our findings to broader or more diverse populations, such as older adults, professionals, or individuals from different cultural or geographical backgrounds. This specific age range was selected for practical reasons, including accessibility and relative consistency in psychological development. Future studies should aim to include a more representative and demographically diverse sample to enhance the applicability of findings.

Second, while our study identifies significant correlations between film genre preferences and personality traits, the cross-sectional nature of the data precludes any claims of causality. It is plausible that preexisting personality traits influence media preferences, rather than film genres shaping personality over time. Longitudinal studies would be essential to investigate potential causal relationships between these variables. Furthermore, future research could explore whether these correlations hold true across various forms of media consumption, such as television or online streaming, to assess whether the genre-personality link extends beyond film.

Additionally, the psychometric properties of the BFI were not formally tested within our sample (e.g., internal consistency or test-retest reliability). While the BFI is a widely validated instrument, this absence may impact the internal validity of our findings and should be addressed in future studies aiming for methodological rigor.

A key implication of our study is the potential application of film genre preferences as a tool in recruitment and screening processes. Understanding how personality traits align with media preferences could offer an additional layer of insight into candidates' psychological profiles. This is particularly relevant in professions that require specific personality characteristics, such as roles that demand high levels of extraversion (e.g., sales or marketing) or conscientiousness (e.g., project management or research). Other practical applications may include educational counseling, where understanding a student's preferences might help personalize learning environments, or digital media platforms tailoring content recommendations based on inferred personality profiles. However, the application of these findings must be approached with caution. It would be necessary to validate the strength and stability of these correlations in more diverse and larger samples before integrating them into professional or clinical settings. Moreover, ethical considerations must be considered when using personal preferences, such as film genres, as part of a candidate assessment tool.

Additionally, comparisons with existing literature suggest that while film preferences can indicate personality traits, they should not be the sole measure used in personality evaluations. Studies have shown that media preferences can be influenced by various factors, including cultural background, mood, and even social context [8,11]. Thus, integrating film genre preferences into recruitment tools would require a more nuanced approach, where these preferences are considered as one part of a broader personality assessment, along with other psychological measures.

In conclusion, this study provides a foundation for further exploration into the relationship between film preferences and personality traits, offering a promising avenue for psychological research and practical applications. However, the generalizability of our results, the potential bidirectional relationship between personality and media preferences, the lack of psychometric validation in this specific sample, and the ethical implications of using film preferences in applied contexts warrant further investigation. Future studies should expand the sample to include diverse populations, employ longitudinal designs to better understand causal links, and explore additional variables that could influence media consumption patterns. By addressing these limitations, future research can help solidify the role of media preferences in personality assessments and their applications in various professional and social contexts.

## Conclusions

This study explored the relationship between college students' preferred film genres and their personality traits as defined by the Big Five theory. The results revealed significant correlations between film genre preferences and personality traits for most genres, except for comedy. Adventure films were positively correlated with extraversion but negatively with conscientiousness and neuroticism, while drama films showed a positive correlation with conscientiousness and neuroticism. Romance films were linked to neuroticism and openness, while horror films were associated with extraversion, agreeableness, and conscientiousness. Mystery films, on the other hand, correlated with openness. These findings suggest that individuals’ film preferences can serve as a reflection of their personality traits, offering a potential tool for personality assessment.

The study also highlights the practical applications of these correlations, particularly in recruitment and screening processes, where film preferences could provide insights into candidates' psychological profiles. These results contribute to the growing body of research on the intersection of media consumption and personality, suggesting that film genre preferences may offer a novel approach to understanding individual differences. While the study is confined to a specific demographic, it opens the door for further research in broader populations and explores how these correlations could be utilized in real-world settings. Ultimately, the study highlights the value of incorporating film preferences as a supplementary tool in personality assessments, particularly in professional contexts.

## Appendices

Questionnaire

Which one of the following is your preferred genre of film?

a. Adventure b. Drama c. Comedy d. Romance e. Horror f. Mystery

Here are a number of characteristics that may or may not apply to you. For example, do you agree that you are someone who likes to spend time with others? Please write a number next to each statement to indicate the extent to which you agree or disagree with that statement.

Disagree strongly: 1 Disagree a little: 2 Neither agree nor disagree: 3 Agree a little: 4 Agree strongly: 5

I see myself as someone who ____.

1. is talkative 2. tends to find fault with others 3. does a thorough job 4. is depressed, blue 5. is original, comes up with new ideas 6. is reserved 7. is helpful and unselfish with others 8. can be somewhat careless 9. is relaxed, handles stress well 10. is curious about many different things 11. is full of energy 12. starts quarrels with others 13. is a reliable worker 14. can be tense 15. is ingenious, a deep thinker 16. generates a lot of enthusiasm 17. has a forgiving nature 18. tends to be disorganized 19. worries a lot 20. has an active imagination 21. tends to be quiet 22. is generally trusting 23. tends to be lazy 24. is emotionally stable, not easily upset 25. is inventive 26. has an assertive personality 27. can be cold and aloof 28. perseveres until the task is finished 29. can be moody 30. values artistic, aesthetic experiences 31. is sometimes shy, inhibited 32. is considerate and kind to almost everyone 33. does things efficiently 34. remains calm in tense situations 35. prefers work that is routine 36. is outgoing, sociable 37. is sometimes rude to others 38. makes plans and follows through with them 39. gets nervous easily 40. likes to reflect, play with ideas 41. has few artistic interests 42. likes to cooperate with others 43. is easily distracted 44. is sophisticated in art, music, and literature

Scoring

Big Five Inventory scale scoring (“R” denotes reverse-scored items):

Extraversion: 1, 6R, 11, 16, 21R, 26, 31R, 36 Agreeableness: 2R, 7, 12R, 17, 22, 27R, 32, 37R, 42 Conscientiousness: 3, 8R, 13, 18R, 23R, 28, 33, 38, 43R Neuroticism: 4, 9R, 14, 19, 24R, 29, 34R, 39 Openness: 5, 10, 15, 20, 25, 30, 35R, 40, 41R, 44

The Big Five Inventory questionnaire and scoring system were obtained from the work by John and Srivastava [17].
